# Functional effects of polymorphisms on glucocorticoid receptor modulation of human anxiogenic substance-P gene promoter activity in primary amygdala neurones

**DOI:** 10.1016/j.psyneuen.2014.04.017

**Published:** 2014-09

**Authors:** Colin W. Hay, Lynne Shanley, Scott Davidson, Philip Cowie, Marissa Lear, Peter McGuffin, Gernot Riedel, Iain J. McEwan, Alasdair MacKenzie

**Affiliations:** a School of Medical Sciences, Institute of Medical Sciences, University of Aberdeen, Aberdeen AB39 3UW, Scotland, UK; b MRC SGDP Centre, Institute of Psychiatry, King's College London, DeCrespigny Park, London SE5 8AF3, UK

**Keywords:** TAC1 promoter, Glucocorticoid receptor, Gene regulation, Single nucleotide polymorphism, Stress and anxiety

## Abstract

Expression or introduction of the neuropeptide substance-P (SP; encoded by the TAC1 gene in humans and Tac1 in rodents) in the amygdala induces anxiety related behaviour in rodents. In addition, pharmacological antagonism of the main receptor of SP in humans; NK1, is anxiolytic. In the current study, we show that the Tac1 locus is up-regulated in primary rat amygdala neurones in response to activation of the glucocorticoid receptor (GR); a classic component of the stress response. Using a combination of bioinformatics, electrophoretic mobility shift assays (EMSA) and reporter plasmid magnetofection into rat primary amygdala neurones we identified a highly conserved GR response sequence (2GR) in the human TAC1 promoter that binds GR in response to dexamethasone (Dex) or forskolin. We also identified a second GR binding site in the human promoter that was polymorphic and whose T-allele is only found in Japanese and Chinese populations. We present evidence that the T-allele of SNPGR increases the activity of the TAC1 promoter through de-sequestration or de-repression of 2GR. The identification of Dex/forskolin response elements in the TAC1 promoter in amygdala neurones suggests a possible link in the chain of molecular events connecting GR activation and anxiety. In addition, the discovery of a SNP which can alter this response may have implications for our understanding of the role of regulatory variation in susceptibility to stress in specific populations.

## 1 Introduction

It is now evident that susceptibility to many forms of human disease is caused by polymorphic variation in the regulatory elements controlling the expression of genes rather than by variation within the coding regions of these genes (Maurano et al., 2012). However, the effects of polymorphic variation on the activity of gene regulatory elements, or their responses to activated signal transduction pathways, has not been widely explored and remains poorly understood.

Substance P (SP), a neuropeptide that, along with the less well characterised neurokinin-A (NKA), is encoded by the human TAC1 gene (Tac1 in rodents) and is expressed in both the medial and central amygdala where it modulates anxiety levels (Ebner et al., 2008; Singewald et al., 2008; Zhao et al., 2009). Stressful stimuli increase levels of SP and Tac1 mRNA within the amygdala regions of restrained rodents (Ebner et al., 2004; Sergeyev et al., 2005) an observation reminiscent of patients diagnosed with stress disorders (Geracioti et al., 2006) or those placed in anxiety inducing situations who have elevated levels of SP in their cerebral spinal fluid (Michelgard et al., 2007). An interesting clue as to the mechanism linking the stress response and the regulation of SP comes from studies that demonstrate co-localisation of the stress activated glucocorticoid receptor (GR) and Tac1 within cells of the central amygdala (Honkaniemi et al., 1992). Interestingly, previous studies on adrenalectomised rats demonstrated a significant up-regulation in Tac1 gene expression in the amygdala of animals treated with corticosterone (Pompei et al., 1995). Glucocorticoids influence gene expression within the brain by acting as ligands to the GR and mineralocorticoid receptors (MR) that subsequently bind DNA to modulate gene expression (Arnett et al., 2010). GR is expressed in the central amygdala but has also been reported in lateral amygdala (Johnson et al., 2005; Prager et al., 2011; Reul and de Kloet, 1985). In addition, selective deletion of GR in the central amygdala has been used to demonstrate its critical role in the formation and consolidation of fear memory (Kolber and Muglia, 2009). Further animal studies have shown that the glucocorticoid and adrenergic systems, the two main stress activated pathways in the brain, act in concert to enhance memory formation by acting within the amygdala, hippocampus and prefrontal cortex (PFC) (van Stegeren et al., 2009).

Given the important role of GR in stress reactions and the anxiogenic properties of SP, surprisingly little is known of how activation of GR might influence TAC1 expression in the amygdala or how these interactions might be affected by polymorphic variation. In the current study, we demonstrate that activated GR can up-regulate the activity of the human TAC1 promoter in primary amygdala neurones and identify the specific sequences involved. We also demonstrate intriguing effects of population specific polymorphisms in the TAC1 promoter on its ability to respond to GR activation. The possible implications of our results on the complex underpinnings of stress and anxiety are discussed.

## 2 Materials and methods

### 2.1 Primary cell culture

Neonate rats were bred internally using wild-type Sprague Dawley rat dams maintained under standard light/dark cycles and given ad libitum access to food and water as outlined in the UK Home Office “Code of practice for the housing and care of animals used in scientific procedure” (1986). Amygdala tissues were dissected from postnatal day 0–3 pups that were humanely sacrificed by cervical dislocation according to UK Home Office Schedule 1 guidelines. Tissues were treated with 0.05% trypsin EDTA (Invitrogen, UK) for 15 min at 37 °C. Trypsin EDTA was replaced with soybean trypsin inhibitor (Sigma, UK) for 5 min at 37 °C to stop the reaction. This was then replaced with supplemented Neurobasal A (Invitrogen, UK) followed by mechanical dissociation. For transfection analysis, cells were then resuspended in culture media [Neurobasal A, B27 (Invitrogen, UK), 1× glutamax (Invitrogen, UK) and Pen/strep (100 U/ml) (Invitrogen, UK)] and plated out at a density of ∼80,000 viable cells/cm^2^ on poly-l-lysine (20 μg/ml) (Sigma, UK) pre-treated plates following cell counting for viable cells using TC10™ automated cell counter (Biorad) with trypan blue. Cells were incubated at 37 °C, 5% CO_2_ for 7 days prior to transfection. For mRNA analysis cells were treated in the same way as above except that they were plated out in six well plates to a density of 500,000 per well and cultured for 5 days prior to treatment with 50 μM Dex for 16 h. RNA was extracted using RNeasy Plus mini kit (Qiagen) and cDNA was synthesised using Applied Biosystems high capacity reverse transcriptase (Life Technologies).

### 2.2 Quantitative RT-PCR

Quantitative RT-PCR to determine Tac1 mRNA expression levels from cultured rat primary amygdala cells treated with either Dex or vehicle was undertaken on derived cDNA using rat qRT-PCR primers (Qiagen) as previously described (Shanley et al., 2010, 2011) using a Roche Light Cycler 480 with Roche SYBR green and normalised using rat TATA binding protein (TBP) specific primers.

### 2.3 Construct production

All derivatives of pTAC1promG-luc that contains the human TAC1 promoter and was previously described as pTAC1*prom-Luc* (Shanley et al., 2010, 2011) were generated using site directed mutagenesis (SDM) using QuickChange II site directed mutagenesis kit (Agilent Technologies, UK). mut2GRTAC1promG-luc was produced using SDM using the following primers 5′-TTGATTTAGAAGAGGGA**CCG**T**A**TGGTTATAGAACGATG-3′ and 5′-CATCGTTCTATAACCA**T**A**CGG**TCCCTCTTCTAAATCAA-3′ (mutated bases in bold see Fig. 2A and B). TAC1promT-luc was produced by SDM using the following oligonucleotide, SNP-SDM 5′-GAAGCAAAAAACGTCCT**T**TTCAACCCCTGCTCCTG-3′ and 5′-CAGGAGCAGGGGTTGAA**A**AGGACGTTTTTTGCTTC-3′ (T-allele in bold see Fig. 2A and D).

### 2.4 Transfections and treatments

All DNA constructs were quantified by spectrophotometery (NanoDrop Technologies) and the quantities of plasmid used for each transfection were adjusted according to the size of each plasmid to ensure molar equivalence between experiments. Firefly luciferase plasmids were co-transfected with Renilla luciferase plasmid, (pGL4.74, Promega, UK), to normalise signals between transfections using magnetic particles (Neuromag, Oz Bioscience, UK) as described in manufacturer's instructions. Primary neuronal cultures were incubated for 24 h prior to agonist or vehicle treatment. Concentrations used were based on previous literature; Dexamethasone (Dex, 50 μM) (Pinho et al., 2011; Poulain et al., 2012; Roomi et al., 2013; Wang et al., 2012), Phorbol 12-myristate 13-acetate (PMA, 160 nM) (Meguro-Horike et al., 2011), forskolin (10 μM) (Szatmari et al., 2007) angiotensin II (AngII, 100 nM) (Modgil et al., 2011). Treatments were dissolved in ethanol (Dex) or DMSO (AngII, forskolin and PMA) and cells exposed for 16 h. Concentrations of DMSO and ethanol were equivalent in study and vehicle control groups and never exceeded 0.05% and 1%, respectively. Cells were harvested and dual luciferase assays were performed as described in manufacturer's instructions using GloMax 96 Microplate Luminometer with dual injectors (Promega, UK).

### 2.5 Nuclear extracts

SH-SY5Y cells were grown in the presence of dexamethasone, forskolin or vehicle (ethanol and DMSO, respectively) for 24 h to a final confluence of approximately 80%. Nuclear extracts were prepared by the method of Dignam et al. (1983) in the presence of protease inhibitors (complete protease inhibitor cocktail from Roche plus 1.0 mM PMSF) and protein phosphatase inhibitors (5 mM β-glycerophosphate and 100 μM activated Na_3_VO_4_).

### 2.6 Electrophoretic mobility shift assays (EMSAs)

Twenty femtomoles of double stranded DNA oligonucleotides, which had been 3′ end-labelled with biotin and HPLC purified, were incubated with either 10 μg SH-SY5Y cell nuclear extracts or 2.0 μM purified full length recombinant human GRα (Thermo Scientific, Cramlington, UK) as previously described (Hay et al., 2005) except that EDTA was replaced with 50 μM ZnSO_4_. The sequences of the oligonucleotides used were: GRE-cons, 5′-TAAGTTTATGGTTACAAACTGTTCTTAAAACGAGG-3′; 2GR, 5′-TTGATTTAGAAGAGGGATGTTCTGGTTATAGAACGATG-3′; 2GR-mut, 5′-TTGATTTAGAAGAGGGACCGTATGGTTATAGAACGATG-3′; SNP-G (SNPGR-G) 5′-GAAGCAAAAAACGTCCTGTTCAACCCCTGCTCCTG-3′; SNP-T (SNPGR-T) 5′-GAAGCAAAAAACGTCCTTTTCAACCCCTGCTCCTG-3′; and a random oligonucleotide containing no regulatory elements (RO) 5′-CGAGCACCCTTCACCCTCCAGGCTTAACGG-3′. The 2GR and SNPGR based oligonucleotides represent human TAC1 promoter sequence. Competing unlabelled oligonucleotides were of the same sequence as the corresponding biotin labelled ones and were added 15 min prior to the addition of labelled probe at 100× molar excess. Supershift analyses were carried out by the inclusion of either 1 μl anti-GR antiserum (Santa Cruz sc1002) or 1 μl pre-immune rabbit serum as a control, and incubation on ice for 30 min prior to the addition of labelled probe. The resulting DNA:protein complexes were resolved on 6% nondenaturing polyacrylamide gels run in 0.5× TBE buffer, pH 8.3 (45 mM Tris-borate, 1 mM EDTA) and visualised using Pierce LightShift Chemiluminescent reagents (Thermo Scientific, Cramlington, UK) according to the manufacturer's protocol. The relative intensities of the DNA:protein complexes were determined by digital integration using a Vilber Loumat Fusion SL cooled CCD sensor with care being taken to ensure that no pixel saturation occurred. Autorads of EMSA gels were used for the figures and the order of some lanes within a gel was altered to aid clarity and facilitate comparisons.

### 2.7 Statistical analysis

Experimental data was derived from tissues recovered from at least three different groups of animals (*n* ≥ 3) and each group was analysed in triplicate. Statistical significance of data sets were analysed using either 2 way ANOVA analysis with post hoc Holm–Sidak test using SigmaPlot Build 11.0.0.75 or one and two tailed Student's *t*-test using GraphPad PRISM version 5.02 where appropriate.

## 3 Results

### 3.1 Treatment of primary amygdala neurones with Dex in culture up-regulates the expression of the endogenous Tac1 gene

**Figure 1:**
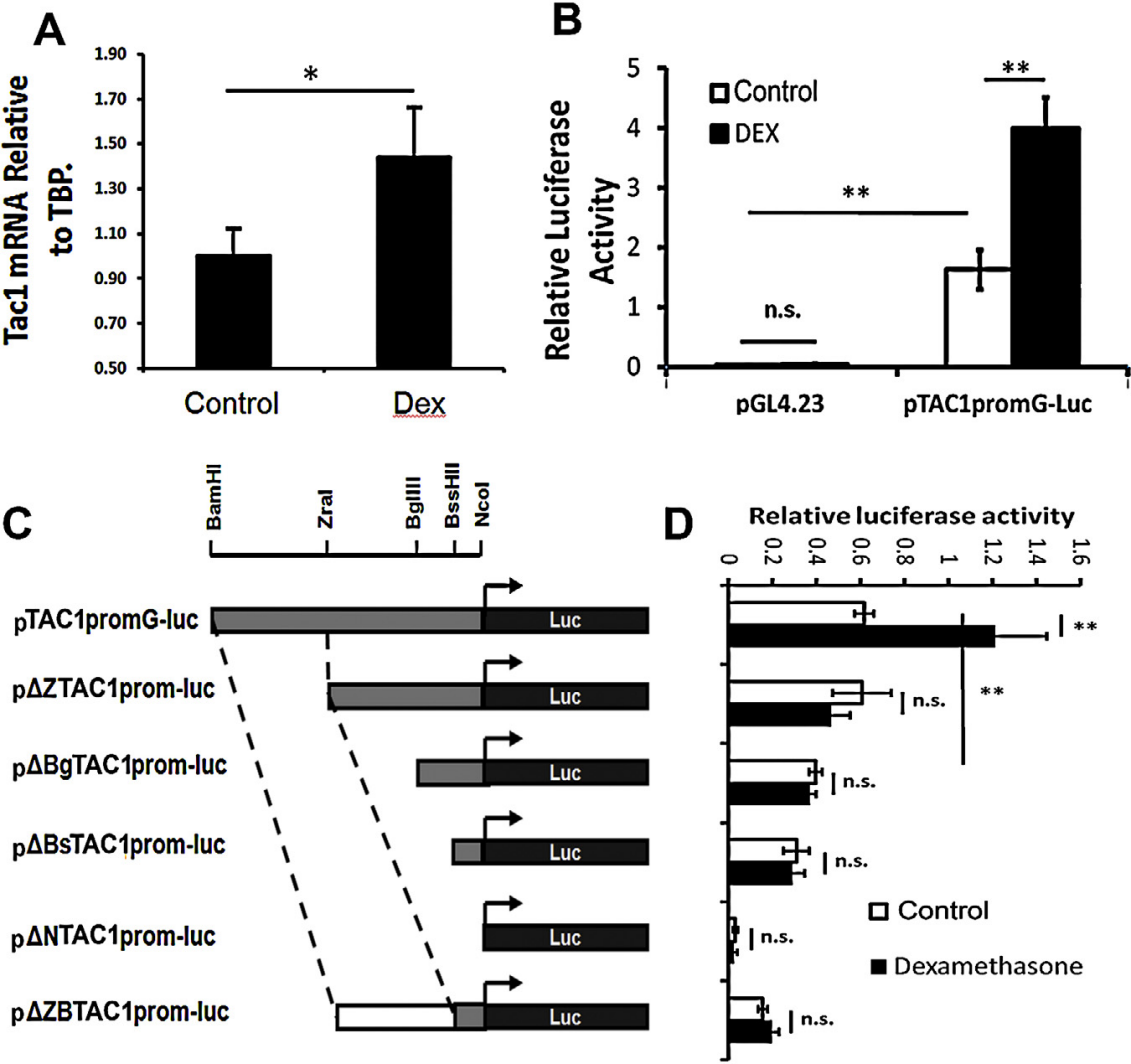
(A) QrtPCR analysis of endogenous Tac1 gene expression in primary rat amygdala cells treated for 16 h with Dex (*n* = 3; *p < 0.05). (B) Relative luciferase gene expression driven by either the minimal promoter construct pGL4.23 or TAC1promG-luc plasmids when transfected into primary amygdala neurones (*n* = 9). (C) Diagrammatic representation (not to scale) demonstrating the linear relationships of the components of each of the different constructs used in the current study. Bent black arrows indicate the transcriptional start site of the luciferase marker gene (Luc). (D) Relative luciferase activity of the constructs represented in C following transfection into amygdala neurones in the presence of vehicle or Dex. In each case *n* > 3. n.s., not significant; *p < 0.05; **p < 0.01.

In order to explore the effects of Dex on the expression of TAC1 in amygdala, primary neonatal rat amygdala neurones (*n* = 3) were cultured for 5 days before being exposed for 16 h to vehicle or Dex. Total RNA was recovered from these cells and qRT-PCR performed on derived cDNA using primers against the rat Tac1 gene and normalised using primers against TBP. Consistently increased levels of Tac1 mRNA of between 25% and 40% were observed following treatment with Dex (Fig. 1A).

### 3.2 The conserved human TAC1 promoter is responsive to Dex in primary amygdala neurones

Because of the known role of TAC1 in anxiety related behaviour and the ability of the Tac1 gene to respond to both stress (Ebner et al., 2004; Sergeyev et al., 2005) and Dex in primary amygdala cell culture, we investigated whether the human TAC1 promoter was responsive to Dex in primary amygdala cells using pTAC1prom-Luc that will be subsequently referred to as pTAC1promG-luc to reflect the specific genotype at the rs17169049 locus (discussed later). pTAC1promG-Luc was magnetofected into primary amygdala neurones and one hour later, cultured in the presence of a vehicle control or Dex. In the absence of stimulation pTAC1promG-luc supported higher levels of luciferase gene expression than the pGL4.23 reporter that contained a minimal TATA box promoter. Significantly, and consistent with data above showing that Dex induced expression of endogenous Tac1, pTAC1promG-luc expression was up-regulated in primary amygdala neurones following 16 h of exposure to Dex (Fig. 1B; *n* = 4; *p* < 0.01).

### 3.3 A 473 bp region at the 5′ end of TAC1prom is required for Dex induction

**Figure 2:**
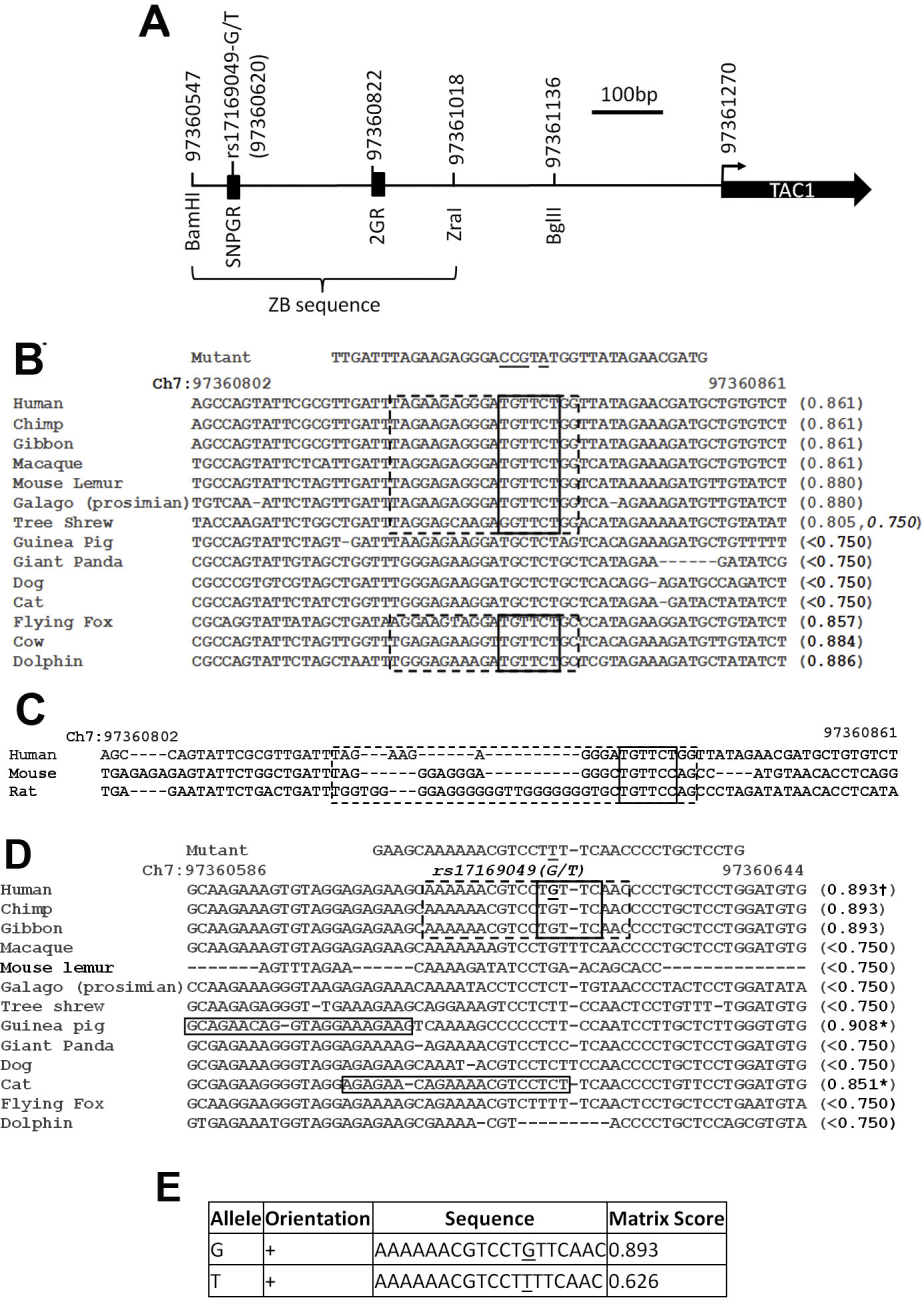
(A) Scale line diagram showing features described in the text including restriction sites, putative GR binding sites (filled black boxes), the rs17169049 SNP and the TAC1 transcriptional start site (bent arrow). Numbering refers to the location of each feature on chromosome 7 based on the Human Feb. 2009 (GRCh37/hg19) assembly. (B–D) Multiple genome alignments of sequences within the TAC1 promoter that contain predicted GR binding sites (B; 2GR and C; SNPGR, binding matrices >0.75 match to Transfac GR consensii) demonstrating alignment between 13 placental mammals. Broken black boxes represent the extent of predicted GR binding matrices and solid black boxes represent matches to known GR core binding motifs. Numbers at the end of each sequence in brackets represent the degree of match to known Transfac GR binding matrices for each species. Numbers with asterisks represent the predicted match of non-homologous GR binding site matrices within the guinea pig and cat TAC1 promoters. ^†^The binding matrix match of the T-allele of rs17169049 is reduced to 0.626. (C) Linear alignment of the human TAC1 promoter sequence around 2GR (broken black box) with the more diverged rat and mouse sequences highlighting the presence of a recognisable GR binding site (Transfac GRQ6 consensus in bold with core sequence highlighted with a solid black box). (B–D) Numbers above the alignments represent the genomic co-ordinates of human sequence (GRCh37/hg19 assembly) and numbers in D in italic represents the position of SNP rs17169049(G/T; underlined in D). Sequences labelled “Mutant” represent the sequence of oligonucleotide primers (changes underlined) used for site directed mutagenesis to produce the mut2GR (B) and the SNPGR-T variant of rs17169049 (D) as well as for the EMSA studies. (E) Table showing readout from RegSNP demonstrating predicted differences (Matrix Score) in GR binding between the G and T alleles of rs17169049.

In order to determine the identity of the Dex response element within the TAC1 promoter, pTAC1promG-luc was subjected to a series of restriction endonuclease deletions that successively removed regions of the TAC1prom region of pTAC1promG-luc (Fig. 1C). These deletion constructs were transfected into amygdala neurones and treated with Dex for 16 h and relative luciferase activities were determined. In the absence of Dex, the removal of a region 473 bp from the 5′ end of the TAC1 promoter region to produce pΔZTAC1prom-luc had no significant effect on basal luciferase expression when compared to pTAC1promG-luc (Figs. 1C and 2A). Deletion of a further 116 bp to produce pΔBgTAC1prom-luc (Figs. 1C and 2A) did, however, reduce basal luciferase expression (Fig. 1C; *n* = 4; *p* < 0.01) and deletion of the entire TAC1 promoter leaving only the transcriptional start site of the luciferase plasmid prevented expression such that no significant difference between the minimal promoter construct (pGL4.23) and pΔNTAC1prom-luc was observed (Fig. 1D; *n* = 3; *p* < 0.01). These data suggest that regulatory elements required to support marker gene expression in amygdala neurones are contained between 219 and 349 bp 5′ of the TAC1 transcriptional start site (between the ZraI and BglII restriction sites; see Figs. 1C,D and 2A). Interestingly, none of the deletion constructs that lacked sequence distal to the ZraI site were responsive to Dex (Figs. 1C,D and 2A). Thus, the regulatory elements required for basal expression in amygdala neurones and those required for the response to Dex are not contained within the same region. Furthermore, Dex response elements are contained within TAC1prom distal to the ZraI restriction site that was termed the ZB sequence (see Figs. 1C and 2A). In order to explore the sufficiency of the ZB sequence for the TAC1prom response to Dex, the ZB sequence was cloned into pGL4.23 that contains a minimal TATA box promoter (pΔNZTAC1G-luc) and transfected into amygdala neurones. However, pΔNZTAC1prom-luc failed to support marker gene expression either in the presence or absence of Dex (data not show). Based on our previous evidence for enhancer-promoter specificity at the TAC1 locus in DRG neurones (Shanley et al., 2010, 2011), we produced a construct containing the ZB sequence next to the smallest region of TAC1prom known to support marker gene expression (pΔBsTAC1prom-luc) to create pΔZBTAC1prom-luc. When transfected into cultured amygdala neurones, pΔZBTAC1prom-luc supported marker gene expression to a level significantly greater than the minimal promoter construct pGL4.23 (*n* = 3; *p* < 0.01) but did not respond to treatment with Dex (Fig. 1C and D). These findings show that the ZB sequence of the TAC1promoter, although required for Dex stimulation of pTAC1promG-luc, is not sufficient and requires the presence of other more proximal sequences.

### 3.4 Identification of a consensus GR site required for Dex induction of pTAC1promG-luc

**Figure 3:**
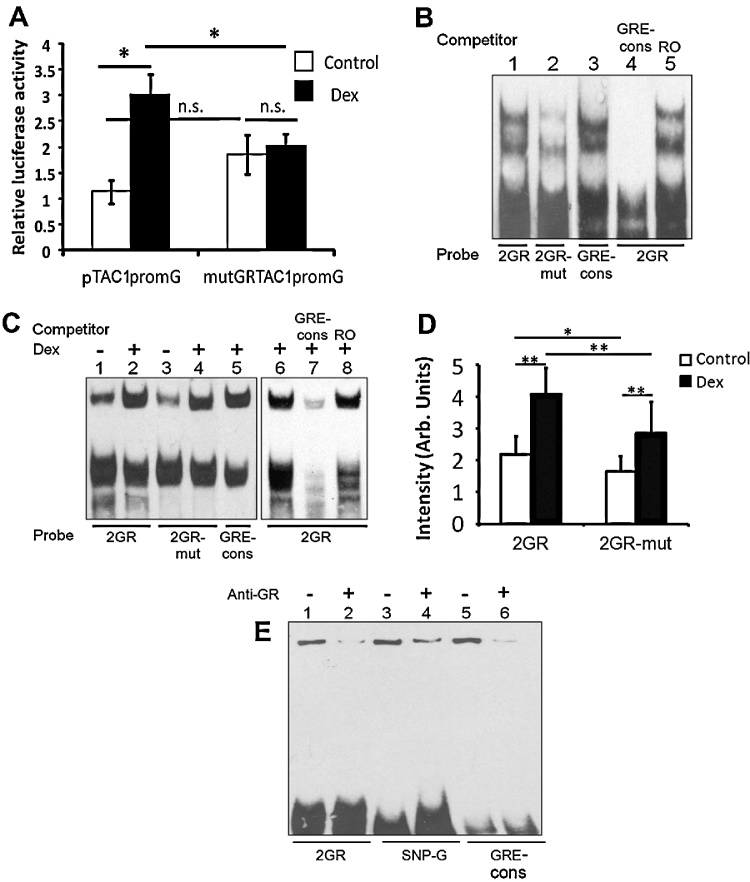
(A) Relative luciferase gene expression driven by either the pTAC1promG-luc or mut2GRTAC1prom-Luc plasmids when transfected into primary amygdala neurones treated with vehicle or Dex (*n* = 9). (B) EMSA analysis of purified recombinant human GRα protein incubated with labelled oligonucleotide probes representing wild type 2GR (2GR); mutated 2GR (2GR-mut) or consensus MMTV-LTR glucocorticoid response element (GRE-cons) and (above) 100 fold molar excess of unlabelled competing GRE-cons oligonucleotides or random oligonucleotide (RO). (C) Lanes 1–6; EMSA analysis of labelled 2GR, 2GR-mut or GRE-cons oligonucleotides incubated with nuclear extracts derived from SH-SY5Y cells treated with vehicle or Dex. Lanes 7 and 8; competitive gel shift assays carried out with competing GRE-cons or RO. (D) Densometric analysis of high molecular weight DNA:protein complexes in lanes 1–4, significance: *p < 0.05; **p < 0.01. (E) *Lanes 1–6*, nuclear extract from SH-SY5Y cells previously incubated in Dex and incubated with labelled probe representing the 2GR, SNP-G or the GR binding consensus sequence (GRE-cons) after pre-incubation with rabbit pre-immune serum (−) or antibody (+). 2GR (Lanes 1 and 2), SNPGR-G (SNP-G; Lanes 3 and 4) and GRE-cons (Lane 5 and 6) were incubated with SH-SY5Y nuclear extracts in the presence or absence of Anti-GR antibody that reduced GR binding to each of the probes. These EMSAs were exposed to electric fields for an additional 30 min to facilitate resolution of high molecular weight DNA:protein complexes.

To further explore the existence of GR response elements (GREs) in the ZB sequence, bioinformatic analysis using Transfac was applied to identify possible GR binding sites. Two GR binding sites were predicted in the human TAC1 promoter. The more proximal, that we called 2GR (ch7:97360822–97360840), lay 448 bp upstream from the TAC1 transcriptional start site (TSS) and was found to be highly conserved in TAC1prom in species as divergent as humans, tree shrews, fruit bats and dolphins (Fig. 2A and B). Although 2GR has been highly conserved in the TAC1 promoter of all other mammalian species rodents demonstrated high levels of divergence within this part of the TAC1 promoter. Despite this, closer analysis demonstrates that the rodent Tac1 promoter has retained a recognisable, although diverged, GR binding site (Fig. 2C). Site directed mutagenesis (SDM) was used to disrupt the core GR binding consensus sequence within the human 2GR to produce a mutant sequence that, following examination of the GR position weight matrices available through Transfac, was less likely to bind GR (Fig. 2B). This construct, mut2GRTAC1promG-luc, was transfected into primary amygdala neurones and cells were treated for 16 h with vehicle control or Dex. Analysis of luciferase gene expression revealed that mutGRTAC1promG was not inducible following treatment with Dex (Fig. 3A; *n* = 4; *p* < 0.05). This demonstrates that the highly conserved consensus 2GR site is required for Dex induction of the TAC1 promoter.

### 3.5 Human recombinant Glucocorticoid receptor protein can bind 2GR

We next reasoned that the GR protein binds directly to the 2GR regulatory element, and examined this possibility by EMSA analysis using purified human recombinant GRα protein. Fig. 3B shows that DNA:protein complexes were formed between purified GRα protein and 2GR oligonucleotide and the pattern was very similar to that seen with consensus GRE oligonucleotide (lanes 1 and 3) which represented the well characterised active consensus GRE (GRE-cons) present in the MMTV-LTR. Using densitometry of three different EMSA experiments we saw that the 2GR-mut oligonucleotide, representing the mutated form of the 2GR, and containing the same mutation as that present in the luciferase reporter plasmids, was bound by GRα with significantly less affinity (*p* < 0.01) than the unmutated 2GR (Fig. 3B, lanes 1 and 2). GRα was completely blocked from binding 2GR by addition of an excess of unlabelled GRE-cons oligonucleotide (Fig. 3B, lane 4) whereas an unlabelled random oligonucleotide had no effect on this interaction (Fig. 3B, lane 5) corroborating high levels of specificity. Further confirmation of GR binding to 2GR was obtained by incubating 2GR and 2GR-mut oligonucleotides in the presence of nuclear extracts derived from a human neuroblastoma cell line, SH-SY5Y, cultured in the presence or absence of Dex (Fig. 3C). Dex treatment significantly increased binding of a single high molecular weight complex to 2GR (Fig. 3C, lanes 1 and 2; *p* < 0.01; *n* = 7). Densitometry analysis of three separate EMSA experiments demonstrated that binding to 2GR-mut was significantly reduced when compared to the un-mutated 2GR in the presence or absence of Dex (Fig. 3D; *n* = 3; *p* < 0.01 and *p* < 0.05, respectively). Addition of an excess of the unlabelled GRE consensus oligonucleotide almost eliminated the Dex inducible complex from the 2GR oligonucleotide (Fig. 3C, lanes 6 and 7) whereas a random oligonucleotide had no effect (Fig. 3C, lane 8). Finally, incubation of Dex treated SH-SY5Y nuclear extract in the presence of serum raised against the GR protein clearly disrupted the interaction of the Dex inducible complex with 2GR (Fig. 3E, lanes 1 and 2).

### 3.6 pTAC1prom-luc also responds to Forskolin

**Figure 4:**
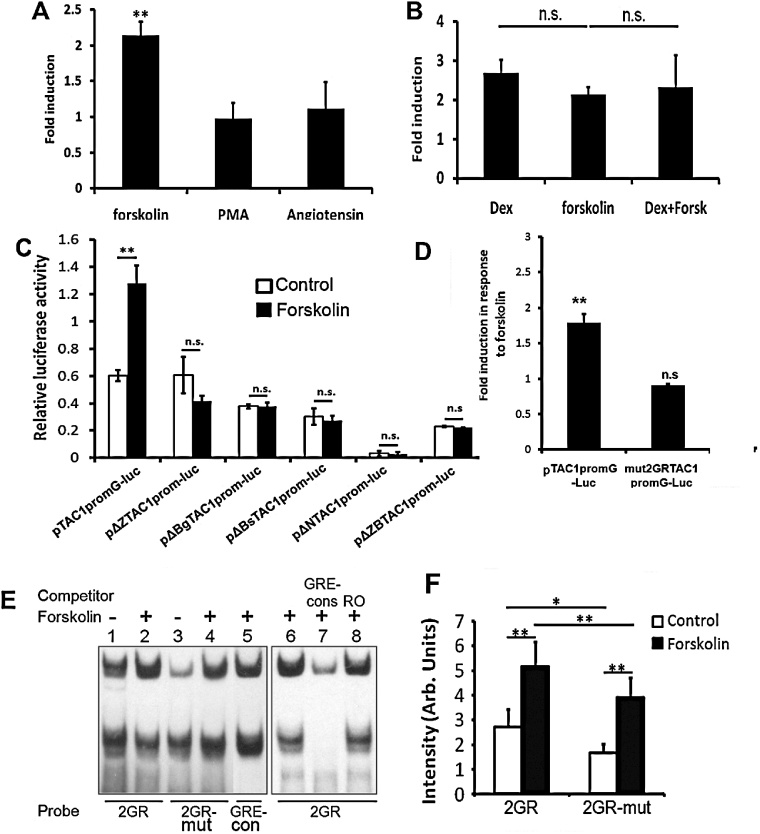
(A) Bar graph representing fold induction of relative luciferase expression following treatment of primary amygdala neurones transfected with pTAC1promG-luc and treated with forskolin, PMA or angiotensin II. (B) Comparison of the fold induction in relative luciferase levels induced from pTAC1promG-Luc in response to treatment with Dex, forskolin or treatment with Dex and forskolin simultaneously. No significant difference was observed between treatments. *n* ≥ 3; *p* = 0.33. (C) Bar graph showing relative luciferase activity in the presence of vehicle or forskolin in each of the constructs represented in Fig. 1D. **p < 0.01; *n* ≥ 3. (D) Bar graph representing relative fold induction of the pTAC1promG-Luc and mut2GRTAC1promG-luc constructs by forskolin. (E) Lanes 1–6; EMSA analysis of labelled 2GR, 2GR-mut or GRE-cons oligonucleotides incubated with nuclear extracts derived from SH-SY5Y cells treated with vehicle or forskolin. Lanes 7 and 8; competitive gel shift assays carried out with competing GRE-cons or RO. (F) Densometric analysis of high molecular weight DNA:protein complexes in lanes 1 to 4, significance: *p < 0.05; **p < 0.01. EMSAs are representative of at least 3 independent experiments and the order of some lanes within a gel has been altered to facilitate comparison.

We next investigated whether stimulation of different intracellular pathways could induce the TAC1 promoter in primary amygdala neurones. In order to explore the identity of other interacting signal transduction pathways amygdala neurones transfected with pTAC1promG-luc were treated with forskolin (adenylate cyclase agonist), PMA (PKC agonist) or AngII (MAPkinase agonist). However, no significant change in expression of pTAC1promG-luc following treatment with either PMA (Fig. 4A; *n* = 4; *p* > 0.05) or AngII (Fig. 4A; *n* = 3; *p* > 0.05) was observed suggesting that neither PKC nor MAPKinase signalling cascades are involved in regulating TAC1prom activity in amygdala neurones. However, treatment with forskolin increased the activity of pTAC1promG twofold (Fig. 4A; *n* = 4; *p* < 0.01). This response was of similar magnitude to that observed following induction with Dex and the response was not altered by simultaneous exposure to Dex and forskolin (Fig. 4B; *p* > 0.05).

### 3.7 2GR is required for TAC1prom response to Forskolin in amygdala neurones

To investigate the elements required for forskolin induced pTAC1promG-luc expression we transfected amygdala neurones with the same deletion constructs used for investigating the identity of the response elements required for Dex induction (Fig. 1C), and treated them with forskolin. In a manner consistent with the results previously obtained for Dex, deletion of the first ZB sequence to produce pΔZTAC1promG-luc or any of the subsequent deletion constructs, removed the ability of TAC1prom to respond to forskolin (Figs. 1D and 4C; *p* > 0.05). These data suggest that the elements required for forskolin induction of pTAC1promG-luc lie in the same region necessary for Dex induction. Furthermore, when transfected into amygdala neurones, mut2GRTAC1prom-G was not induced following treatment with forskolin (Fig. 4D; *n* = 5; *p* < 0.01). Thus, consistent with the results obtained for Dex, the 2GR site is essential for forskolin-induced expression of pTAC1promG-luc.

### 3.8 2GR binds GR in the presence of forskolin

We explored whether binding of GR to 2GR could also be stimulated by forskolin as PKA activation is known to influence GR binding (Gruol and Altschmied, 1993; Gruol et al., 1986). EMSAs were carried out on nuclear extracts derived from SH0SY5Y cells treated with vehicle or forskolin. Cell extracts derived from cells exposed overnight to forskolin contained GR proteins with increased ability to bind 2GR (Fig. 4E(lanes 1 and 2) and F; *n* = 3; *p* < 0.01). While forskolin was still capable of significantly inducing GR binding to 2GRmut its ability to do so was significantly reduced when compared to 2GR (Fig. 4E(lanes 1–4) and F; *n* = 3; *p* < 0.01).

### 3.9 Identification of a second polymorphic consensus GR binding site within TAC1prom

In addition to 2GR, bioinformatic analysis of the Dex responsive ZB sequence also predicted the presence of a second consensus GR binding site 643 bp away from the TAC1 TSS which was termed SNPGR (Fig. 2A and D). In contrast to 2GR, which was highly conserved in many different mammalian species, SNPGR is only conserved in higher primates (Fig. 2D). Interestingly, SNPGR contained a single nucleotide polymorphism (SNP; rs17169049; G-T) whose minor T-allele has only been described in Chinese and Japanese populations where 12% and 20% of these populations are heterozygous respectively.

### 3.10 The T-allele of rs17169049 within SNPGR binds GR to a lesser extent than G allele

**Figure 5:**
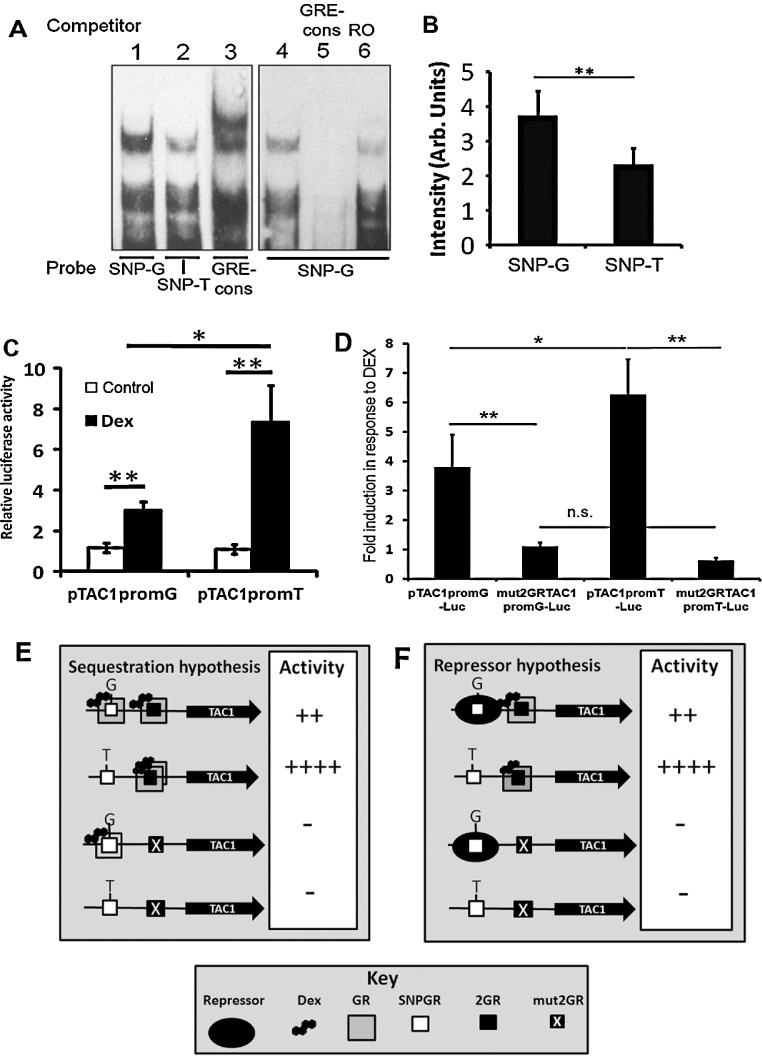
(A) EMSA analysis of purified recombinant human GRα protein incubated with oligonucleotide probes representing the T or G alleles of the SNPGR regulatory element (SNP-G and SNP-T) or consensus GRE (GRE-cons). (B) Densometric analysis of high molecular weight DNA:protein complexes in lanes 1 and 2 (*n* = 3; ** *p* < 0.01). (C) Relative luciferase gene expression driven by either the pTAC1promG-luc or pTAC1promT-Luc plasmids when transfected into primary amygdala neurones treated with vehicle or Dex (*n* = 9). (D) Bar graph representation of the fold induction in relative luciferase expression from the indicated luciferase reporter constructs transfected into primary amygdala neurones in response to overnight treatment of primary amygdala neurones with Dex compared with vehicle treated samples following magnetofection with SDM altered versions of pTAC1prom-Luc. In each case *n* > 3. **p < 0.01; *p < 0.05. (E and F) Diagrammatic representations of the two different hypotheses presented in the discussion to explain the effects of the T-allele of SNPGR on the activity of 2GR. (E) The sequestration hypothesis and (F) The repression hypothesis.

Using the RegSNP programme (viis.abdn.ac.uk/regsnp/Home.aspx) we predicted that GR binding to the T-allele of rs17169049 within SNPGR (SNPGR-T) would be reduced by 26.7% compared to the major G allele (SNPGR-G; Fig. 2D). In order to explore this prediction we incubated purified GRα protein in the presence of oligonucleotides corresponding to both allelic variants of the SNPGR sequence and analysed their interactions using EMSA (Fig. 5A). Intriguingly, densometric analysis of the high molecular weight DNA:protein complexes in 3 different EMSAs showed that binding of GRα to the SNPGR-T was 37% less when compared with binding to SNPGR-G (Fig. 5A(lanes 1 and 2) and B; *n* = 3; *p* < 0.01). Excess unlabelled GRE-consensus oligonucleotide could completely disrupt GRα binding to SNPGR demonstrating the specificity of the interaction whereas a random oligonucleotide (RO) had no effect (Fig. 5A, lanes 4–6). In addition, binding of GR to SNPGR-G was corroborated by incubation of Dex treated SH-SY5Y nuclear extract in the presence of serum raised against the GR protein which disrupted DNA:protein complex formation (Fig. 3E, lanes 3 and 4).

### 3.11 The SNPGR-T allele of TAC1prom is more responsive to Dex induction

To determine whether the different alleles of SNPGR play a role in altering tissue responsiveness to Dex, site directed mutagenesis of pTAC1promG-luc was used to produce pTAC1promT-luc that contained SNPGR-T. Exposure of amygdala neurones transfected with pTAC1promT-luc to Dex for 16 h led to a significant increase in luciferase activity compared to vehicle treated neurones transfected with pTAC1promT-luc (Fig. 5C; *n* = 5; *p* < 0.01). Despite no difference in luciferase activity between the two alleles in the absence of Dex (Fig. 5C; *p* > 0.05), TAC1promT-luc responded to Dex treatment much more strongly than the major allele TAC1promG-luc (Fig. 5C; *n* = 5; *p* < 0.05).

### 3.12 Both alleles of SNPGR are unresponsive to Dex induction in the absence of 2GR

We examined the role of the SNPGR site in the TAC1prom response to Dex by mutating the 2GR sites from both TAC1promG and TAC1promT. In both cases, neither the G or T alleles of the TAC1promoter responded to Dex in the presence of the mutated 2GR site (Fig. 5D). These results suggest that, although both SNPGR alleles were able to bind GR to varying degrees (Fig. 5A, lanes 1 and 2), these interactions were insufficient to activate transcription of TAC1prom in the absence of 2GR and in the presence of Dex.

## 4 Discussion

In the current study, we demonstrate that activation of GR is able to increase Tac1 mRNA expression in cultured rat primary amygdala neurones. Using a combination of reporter construct deletion analysis and gene magnetofection into primary amygdala neurones, we show that a 723 bp fragment of the TAC1 promoter is highly Dex inducible and that the most distal 473 base pairs of this fragment are necessary for this response. Further analysis using bioinformatics and comparative genomics predicted the presence of a highly conserved GR binding site. This site was termed 2GR, and its mutation prevented TAC1prom from responding to GR activation in primary amygdala neuronal cultures. EMSA analysis with purified GR protein revealed an interaction between 2GR and the GR protein, which could be disrupted using an unlabelled consensus GR oligonucleotide. Finally the incubation of Dex-treated SH-SY5Y cell nuclear extracts with anti-GR antibody greatly reduced the binding of the Dex induced complex to 2GR. These separate lines of evidence corroborate the hypothesis that GR activation can modulate TAC1 gene expression in amygdala at the highly conserved 2GR sequence.

In addition to glucocorticoids, the stress response involves the production of epinephrine that binds and activates adrenergic receptors that influence gene expression via the PKA pathway (Deyama et al., 2008). We demonstrated that TAC1prom activity was responsive to forskolin activation in primary amygdala neurones; a response that depended on the 2GR binding site. These observations support the hypothesis that, in addition to Dex, binding of GR to 2GR can also be modulated by PKA activation. Our observations are consistent with previous reports that forskolin and cAMP analogues such as 8-bromo-cAMP and dibutyryl-cAMP, increased glucocorticoid steroid binding to GR (Gruol and Altschmied, 1993; Gruol et al., 1986; Oikarinen et al., 1984). Analysis of the pathways controlling the expression of proenkephalin (ProENK) and thyroid releasing hormone genes also identified an interaction between glucocorticoid and PKA pathways that, in the case of ProENK, did not involve CREB or AP-1 phosphorylation (Perez-Martinez et al., 1998; Won and Suh, 2000). Further studies demonstrated a functional interaction between PKA pathways and glucocorticoids within the amygdala in vivo (Roozendaal et al., 2002). These observations suggest that the GR influence on TAC1prom activity may be modified by parallel induction through the PKA and glucocorticoid pathways providing a combinatorial mechanism through which corticosteroids and epinephrine may alter the expression of the TAC1 gene in amygdala neurones. It has not escaped our notice that the discovery of a highly conserved sequence within the human TAC1 promoter that responds to GR activation provides a critical “link” in the “chain” of events that links the stress response (through GR activation) to anxiety (through up-regulation of TAC1 expression in the amygdala). If a way could be found to manipulate this interaction in the future it may be possible to modulate the mechanistic link between stress and anxiety and reduce the worst symptoms of conditions such as post-traumatic stress disorder.

It is also important to consider that, in addition to SP, TAC1 also encodes another neuropeptide called neurokinin-A (NKA) which, because it is encoded by the same mRNA, is also likely to be expressed in the amygdala and to be affected by GR modulation of the TAC1 promoter. This is interesting as antagonist blockade of the NKA receptor; NK2, using saredutant (SR48968) has demonstrated anti-depressant-like activity in several rodent models (Louis et al., 2008) and provides a second possible mechanistic link between GR activation and mood modulation through expression of TAC1.

This model is, however, further complicated by the presence of a second putative GR binding site in humans 193 base pairs distal of 2GR. This second GR site is less conserved than 2GR and contains a polymorphism (rs17169049-G/T) that has so far only been observed in Japanese and Chinese populations. Using the RegSNP on-line algorithm we predicted that the less frequent T-allele of SNPGR would have 26.7% less affinity for GR than the more common G-allele. These predictions were confirmed using EMSA studies which showed that the T-allele reduced binding of GR in the form of purified protein to SNPGR by 37%. Based on this observation we predicted that the TAC1promoter containing the G-allele of SNPGR would demonstrate greater sensitivity to Dex by working in tandem with 2GR. However, we were surprised to find that the T-allele of TAC1prom was twice as strongly induced as was the G-allele. Furthermore, mutation of 2GR within the TAC1prom reporter showed that, despite being able to bind GR, neither SNPGR-G nor SNPGR-T is able to contribute to the response of TAC1prom to Dex. Clues as to how this polymorphism affects TAC1 promoter GR response come from experiments that have used “decoy” oligonucleotides to manipulate the activity of promoter elements in cells (Obata et al., 2011). Using this approach activated transcription factors are sequestered away from promoter regions following transfection of “decoy” oligonucleotides. A number of studies have suggested that variation within repetitive elements may affect the activity of promoters by sequestering activated transcription factors (Lee and Maheshri, 2012; Liu et al., 2007). Based on these studies, we hypothesise that SNPGR may act as a “decoy” site for activated transcription factors such as GR. Thus, SNPGR-G may sequester GR proteins from the functional 2GR site thereby reducing the GR-mediated induction of TAC1prom. In contrast SNPGR-T is less able to sequester GR thus increasing the likelihood of GR binding to 2GR (Fig. 5E). A competing, though less elegant, hypothesis is that the SNPGR-G allele binds a repressor protein that reduces the affinity for GR binding at 2GR to activate RNApolII whereas the T-allele lacks affinity for said repressor (Fig. 5F).

Clearly, more work is required to identify the precise mechanisms that allow the SNPGR-G allele to reduce the ability of 2GR to respond to GR activation. It will also be of intense interest to discover how the activation of both GR and MR by corticosterone influences the activity of the TAC1 promoter. Moreover, the phenomenon of allele specific repression of GR activation outlined here is significant for our understanding of disease mechanisms; suggesting that SNPs within demonstrably inactive parts of the genome can significantly affect the activity of functional elements.

Anxiety disorders have been diagnosed in all human societies, and display cultural particularities in symptoms and prevalence. Although the observed differences in rates among the various disorders across cultural groups reflect dissimilar prevalence, variability in measurement equivalence, diagnostic criteria and precision are also factors (Lewis-Fernandez et al., 2010) and preclude simple attempts to determine a direct correlation between the SNPGR genotype and general anxiety levels. However, now that the biological implications of this SNP have been revealed in this report, it is vital that research groups with the necessary resources e.g. DNA libraries from subjects of different ethnicities with accurate psychological assessments, explore possible phenotypic consequences. A tantalising suggestion of the potential importance of this SNP comes from examination of the haplotype frequencies in the publically available dbSNP database (http://www.ncbi.nlm.nih.gov/SNP/). The observed haplotype frequencies for G/G homozygotes and G/T heterozygotes in the Japanese and Han Chinese groups adhere to Hardy-Weinberg proportions, however, no T/T homozygote was observed. Intriguingly, the T allele frequency and sample number (*n* = 170) in the Japanese study were both sufficiently high that the absence of T/T homozygotes was of statistical significance (*p* < 0.05).

It is likely that the ability of TAC1prom to respond to GR activation arose early in mammalian evolution to allow the up-regulation of TAC1 expression in the amygdala in response to stressful stimuli such as predation, disease, famine or drought. However, the more recent evolution of SNPGR may have allowed a tempering of the GR response of TAC1prom in higher primates possibly serving to reduce the effects of stress hormones on the expression of TAC1 and reducing levels of anxiety in these animals. Moreover, the accumulation of SNPGR-T in modern Japanese and Chinese populations is of particular interest in light of the disturbing rates of suicide reported by the World Health Organisation in this part of the world (Kohyama, 2011). Expanded population studies should cast light on the possible role of the SNP, and have the potential to facilitate development of personalised therapeutic strategies.

## Role of the funding sources

All the funding sources listed in the acknowledgements were used to pay for consumables, equipment, animals and salaries. Marissa Lear and Lynne Shanley were funded by the Wellcome Trust (080980/Z/06/Z). Scott Davidson was funded by a BBSRC strategic studentship (BBS/S/2005/12001). The BBSRC (BB/D004659/1) and by Medical Research Council (G0701003). Colin Hay was funded by the Chief Scientist Office, Scotland. Philip Cowie was funded by the Scottish Universities Life Science Alliance (SULCA).

## Conflict of interest

The authors declare no conflict of interest.

## Contributors

Colin W. Hay, Lynne Shanley, Scott Davidson, Philip Cowie, Marissa Lear all carried out work and data analysis that directly contributed to this manuscript. Peter McGuffin, Gernot Riedel, Iain J. McEwan, and Alasdair MacKenzie all contributed to the analysis of the data and the drafting of the final manuscript and figures.
